# Association of XPC Polymorphisms and Lung Cancer Risk: A Meta-Analysis

**DOI:** 10.1371/journal.pone.0093937

**Published:** 2014-04-15

**Authors:** Bo Jin, Yu Dong, Xueyan Zhang, Huimin Wang, Baohui Han

**Affiliations:** Department of Pulmonary Medicine, Shanghai Chest Hospital, Shanghai Jiaotong University, Shanghai, China; University of Birmingham, United Kingdom

## Abstract

**Background:**

Xeroderma pigmentosum complementation group C gene (XPC) is a key member of nucleotide excision repair pathway and plays an important role in human DNA repair system. It is reported that several common polymorphisms of XPC are associated with susceptibility to lung cancer. However, the conclusion is still elusive.

**Method:**

This meta-analysis was performed to determine the relationship between XPC polymorphisms (Lys939Gln, Ala499Val, and PAT) and lung cancer risk. Published literatures were identified by searching online databases and reference lists of relevant studies. Odds ratios (ORs) and 95% confidence intervals (CIs) were calculated to estimate the association strength. Publication bias were detected by Egger’s and Begg’s test.

**Result:**

After strict screening, we identified 14 eligible studies in this meta-analysis, including 5647 lung cancer cases and 6908 controls. By pooling all eligible studies, we found that the homozygote Gln939Gln genotype was associated with a significantly increased risk of lung cancer in Asian population (GlnGln vs LysLys, OR = 1.229, 95% CI: 1.000–1.510; GlnGln vs LysLys/LysGln, OR = 1.257, 95% CI: 1.038–1.522). As for the PAT polymorphism, in Caucasian population, we found carriers of the −/− genotype were associated significantly reduced risk of lung cancer in homozygote comparison model (−/− vs +/+, OR = 0.735, 95% CI: 0.567–0.952).

**Conclusion:**

In this meta-analysis we found that Gln939Gln genotype was associated with significantly increased risk of lung cancer in Asian population; the PAT −/− genotype significantly reduced susceptibility to lung cancer in Caucasian population; while the XPC Ala499Val polymorphism was not associated with lung cancer risk.

## Introduction

Lung cancer is the leading cause of cancer-related death worldwide. In 2008, 1.61 million new cases of lung cancer were diagnosed, accounting for 12.7% of all new cases of malignant tumors [1]. Although smoking has been demonstrated as a predominant risk factor of lung cancer, only a small proportion of smokers developed lung cancer during their lifetime [2]. The individual susceptibility to lung cancer can be partially explained by genetic variation [3] and possible gene-environment interactions [4].

A lot risk factors, such as tobacco smoking, ultraviolet, and ionizing radiation, can cause the formation of bulky adducts, crosslinks, and strand breaks in DNA [2]. These DNA damages are repaired by four major DNA repair pathways, namely nucleotide excision repair (NER), base excision repair (BER), double strand break repair (DSBR) and mismatch repair (MMR) pathways [5]. More than a hundred protein coding genes involved in human DNA repair system pathways have been identified and studies showed that single nucleotide polymorphisms in multiple DNA repair-related genes are associated DNA repair capacity [6], [7]. Cigarette smoking caused DNA damage is mainly repaired by the NER pathway [8]. A lot of studies have shown that several polymorphisms of xeroderma pigmentosum complementation group C gene (XPC), a key enzyme in the NER pathway, are associated with impaired DNA repair capacity and susceptibility to lung cancer [1], [9]–[12]. Although more than a hundred SNPs in the coding regions of XPC have been reported, two common SNPs were most investigated: the Lys939Gln (rs2228001) polymorphism in the domain interacting with TFIIH, and the Ala499Val (rs2228000) polymorphism in the domain interacting with RAD23B. Additionally, the XPC intronic poly-AT insertion/deletion polymorphism (PAT) was also associated with lung cancer risk in Caucasians [13]. However, the association between XPC polymorphisms (Lys939Gln, Ala499Val, and PAT) and lung cancer risk was still inconclusive and previous meta-analyses did not fully elucidate this issue [11], [12]. Thus, we performed this update meta-analysis to provide more precise estimation of the relation between XPC polymorphisms and susceptibility to lung cancer.

## Methods

### Literature Searching

This meta-analysis was performed and reported according to the PRISMA guideline (Checklist S1). A literature search was carried out using PubMed, EMBASE and China National Knowledge Infrastructure (CNKI) database up to November 2013. There were no restriction of origin or languages. Searching terms included combinations of medical subheadings and key words of “Xeroderma Pigmentosum, Complementation Group C” or “XPC”, “polymorphisms, single nucleotide” or “SNP”, and “neoplasm, lung” or “lung cancer”. Other alternative spellings and abbreviations were also considered. The reference lists of previous meta-analyses were manually examined to identify additional relevant studies.

### Inclusion and Exclusion Criteria

Studies were selected according to the following inclusion criteria: (1) full-text published articles; (2) epidemiological association studies with a hospital-based or population-based design; (3) investigating the association between XPC polymorphisms (Lys939Gln, Ala499Val, and PAT) and lung cancer risk; (4) providing detail genotype frequencies for calculating pooled odds ratio. The exclusion criteria were as follows: (1) studies without detail genotype frequencies, which were unable to calculate odds ratio; (2) if there were multiple reports of the same study, only the one with most participants or the most recent one was included and the others were excluded. Titles and abstracts of searching results were screened and full text papers were further evaluated to confirm eligibility. Two reviewers (Bo Jin and Yu Dong) independently selected eligible studies. Disagreement between the two reviewers was settled by discussing with the third reviewer (Baohui Han).

### Data Extraction

The following data was collected by two reviewers (Bo Jin and Yu Dong) independently using a pre-designed form: name of first author, publishing time, country where the study was conducted, genotyping methods, ethnicity, source of control, number of cases and controls, genotype frequency in cases and controls. Studies with a sample size of more than 500 participants were defined as “large”; otherwise “small”. Different ethnicity descents were categorized as Asian and Caucasian. Eligible studies were defined as hospital-based (HB) and community-based (PB) according to the source of control.

### Statistical Analysis

The association strength between XPC polymorphisms (Lys939Gln, Ala499Val, and PAT) and cancer risks was measured by odds ratio (OR) with 95% confidence intervals (95% CI). The estimates of pooled ORs were achieved by calculating a weighted average of OR from each study. A 95% CI was used for statistical significance test and a 95% CI without 1 for OR indicating a significant increased or reduced cancer risk. Odds ratios of 5 comparison models were calculated: homozygote (AA vs. aa), heterozygote (Aa vs. aa), dominant (AAAa vs. aa), recessive (AA vs. Aaaa), and allele (A vs. a) comparison models (A, variant allele; a, wild allele; the XPC 939Gln, 499Val, and PAT – alleles were assumed as variant alleles). subgroup analyses were performed according to (i) source of control, (ii) ethnicities, and (iii) sample size, to examine the impact of these factors on the association. To test the robustness of association and characterize possible sources of statistical heterogeneity, sensitivity analysis were carried out by excluding studies one-by-one and analyzing the homogeneity and effect size for all of rest studies.

Chi-square based Q test was used to check the statistical heterogeneity between studies, and the heterogeneity was considered significant when p<0.10 [14]. The fixed-effects model (based on Mantel-Haenszel method) and random-effects model (based on DerSimonian-Laird method) were used to pool the data from different studies. The fixed-effects model was used when there was no significant heterogeneity; otherwise, the random-effects model was applied[15]. Publication bias was assessed using Begg’s test and Egger’s test[16]. HWE (Hardy-Weinberg equilibrium) was tested by Pearson’s X^2^ test (P<0.05 means deviated from HWE). All analyses were performed using Stata version 11.0 (StataCorp, College Station, TX).

## Results

**Figure 1:**
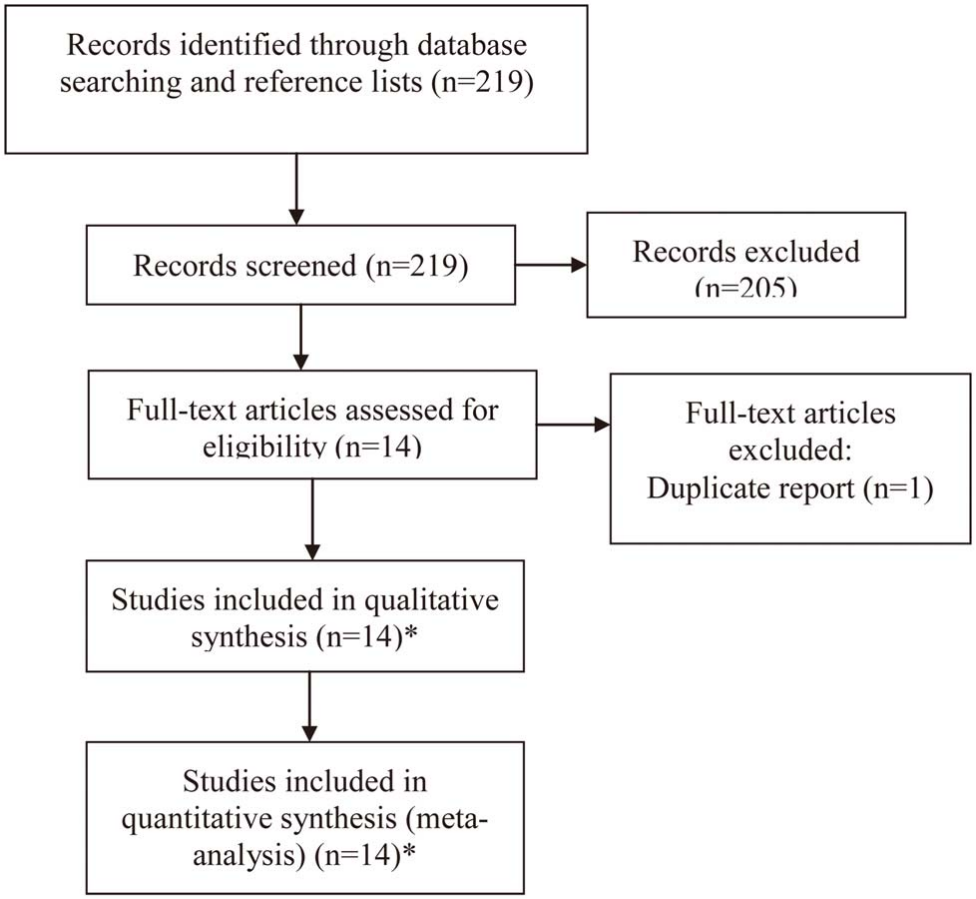
Flow Diagram of study selection. *the two populations in Chang JS’s study were treated as 2 separate studies.

By searching online databases and references and related papers, 219 records were retrieved. After the primary screening of titles and abstracts, 14 full-text articles were identified [1], [9], [10], [13], [17]–[26], one [26] of which was excluded for the reason of duplicate reports from one study (Figure 1). In the study reported by Chang JS [10], two separate population was included and the data were represent independently; thus, each population was treated as an independent study. Therefore, a total of 14 eligible studies were included and analyzed in this meta-analysis[1], [9], [10], [13], [17]–[25], including 5647 lung cancer cases and 6908 controls.

The Lys939Gln polymorphism was investigated in 10 studies [1]–[3], [10], [12], [21], [23], [24] and most of them were performed in Caucasian population. The Ala499Val polymorphism was reported in 5 [9], [12], [20], [21], [24]studies and 4 of them were conducted in Asian population. And 5 studies[3], [12], [15], [16], [25] investigated the association between PAT polymorphism and lung cancer risk. Of the 14 eligible studies, 6 studies were hospital-based and 7 studies used PCR-RFLP method. Except the Lys939Gln polymorphism reported by Shen M[21], all genotype distribution data of XPC polymorphisms were in accordance with HWE. The baseline characteristics of eligible studies were shown in table 1.

**Table 1:** Baseline Characteristics of Eligible Studies.

Author	Year	Country	Ethnicity	Control	HWE	Genotyping	Polymorphisms	Cases	Controls
Letkova L	2013	Slovak Republic	Caucasian	HB	YES	PCR-RFLP	Lys939Gln	382	379
Sakoda LC	2012	USA	Caucasian	CB	YES	Golden Gate	Ala499Val,Lys939Gln	744	1477
Chang JS^a^	2011	USA	Caucasian	CB	YES	Golden Gate	Lys939Gln	368	579
Raaschou-Nielsen O	2009	Denmark	Caucasian	CB	YES	RT-PCR	Lys939Gln	430	790
López-Cima MF	2007	Spain	Caucasian	HB	YES	PCR-RFLP	PAT	516	533
De Ruyck K	2007	Belgium	Caucasian	HB	YES	PCR-RFLP	PAT	110	110
Bai Y	2007	China	Asian	HB	YES	TaqMan	Ala499Val,Lys939Gln	1010	1011
Hu ZB	2005	China	Asian	CB	YES	PCR-RFLP	Ala499Val,Lys939Gln	320	322
Shen M	2005	China	Asian	CB	No^b^	RT-PCR	Ala499Val,Lys939Gln	122	122
Vogel U	2005	Denmark	Caucasian	CB	YES	RT-PCR	Lys939Gln	267	269
Lee GY	2005	Korea	Asian	HB	YES	PCR-RFLP	Ala499Val,Lys939Gln,PAT	432	432
Marín MS	2004	Spain	Caucasian	HB	YES	PCR-RFLP	PAT	359	375
Wang YG	2003	China	Asian	CB	YES	PCR-RFLP	PAT	597	509

a the two populations were treated as two separate studies; b: disagreement of HWE for Lys939Gln polymorphism; HB: hospital-based studies; CB: community-based studies.

### XPC Lys939Gln Polymorphism

**Figure 2:**
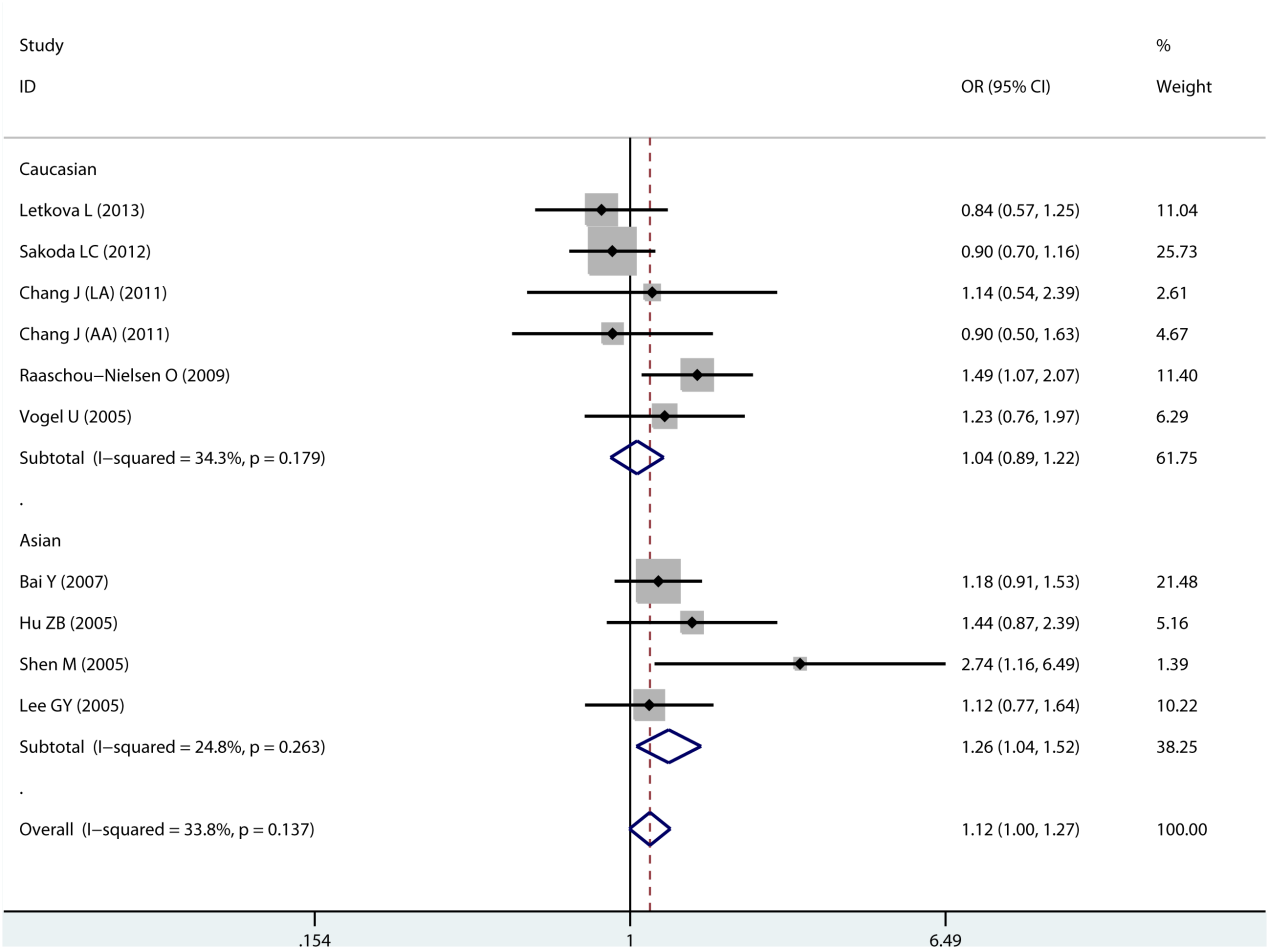
Forrest plot of XPC Lys939Gln polymorphism (GlnGln vs. LysLys/LysGln) by ethnicity.

4030 lung cancer cases and 5336 controls were available for the analysis of XPC Lys939Gln polymorphism and the meta-analysis results were showed in table 2. In overall analysis, no significant association of XPC Lys939Gln polymorphism with lung cancer risk was observed in any of the 5 comparison models. Sub-group analysis showed that source of controls and sample size did not alter the association. However, in the subgroup analysis according to ethnicity, we found that the homozygote Gln939Gln genotype was associated with a significantly increased risk of lung cancer in Asian population (GlnGln vs LysLys, OR = 1.229, 95% CI: 1.000–1.510; GlnGln vs LysLys/LysGln, OR = 1.257, 95% CI: 1.038–1.522; Figure 2), while no association was found among Caucasian population. Meta-analysis results for Lys939Gln polymorphism were shown in Table 2. No evidence of publication bias was detected by Begg’s test (P = 0.283, Figure S1) and Egger’s test (P = 0.186).

**Table 2:** Meta-analysis results of XPC Lys939Gln polymorphism.

	Homozygote Model		Heterozygote Model		Dominant Model		Recessive Model		Allele Model	
	OR(95% CI)	Heterogeneity	OR(95% CI)	Heterogeneity	OR(95% CI)	Heterogeneity	OR(95% CI)	Heterogeneity	OR(95% CI)	Heterogeneity
Lys939Gln										
Overall	1.123(0.985–1.281)	0.222	0.994(0.908–1.087)	0.309	1.021(0.938–1.112)	0.32	1.124(0.996–1.269)	0.137	1.693(0.986–2.907)	0.227
HB	1.068(0.871–1.309)	0.474	0.976(0.847–1.125)	0.279	0.997(0.871–1.140)	0.358	1.080(0.895–1.303)	0.366	1.018(0.926–1.120)	0.483
CB	1.165(0.981–1.384)	0.127	1.006(0.895–1.130)	0.247	1.038(0.929–1.159)	0.228	1.157(0.988–1.356)	0.08	1.056(0.976–1.143)	0.126
Caucasian	1.056(0.890–1.253)	0.228	1.012(0.899–1.139)	0.231	1.021(0.913–1.142)	0.207	1.042(0.890–1.220)	0.179	1.021(0.943–1.106)	0.145
Asian	1.229(1.000–1.510)*	0.283	0.969(0.843–1.113)	0.326	1.021(0.895–1.165)	0.362	1.257(1.038–1.522)*	0.263	1.069(0.972–1.175)	0.385
Large	1.097(0.959–1.256)	0.259	0.983(0.896–1.079)	0.449	1.007(0.922–1.100)	0.369	1.100(0.972–1.246)	0.225	1.028(0.966–1.095)	0.233
Small	1.763(0.983–3.163)	0.381	1.160(0.812–1.657)	0.085	1.255(0.892–1.766)	0.25	1.693(0.986–2.907)	0.13	1.265(0.985–1.624)	0.909

OR: odds ratio; CI: confidence intervals; *significant association.

### XPC Ala499Val Polymorphism

**Figure 3:**
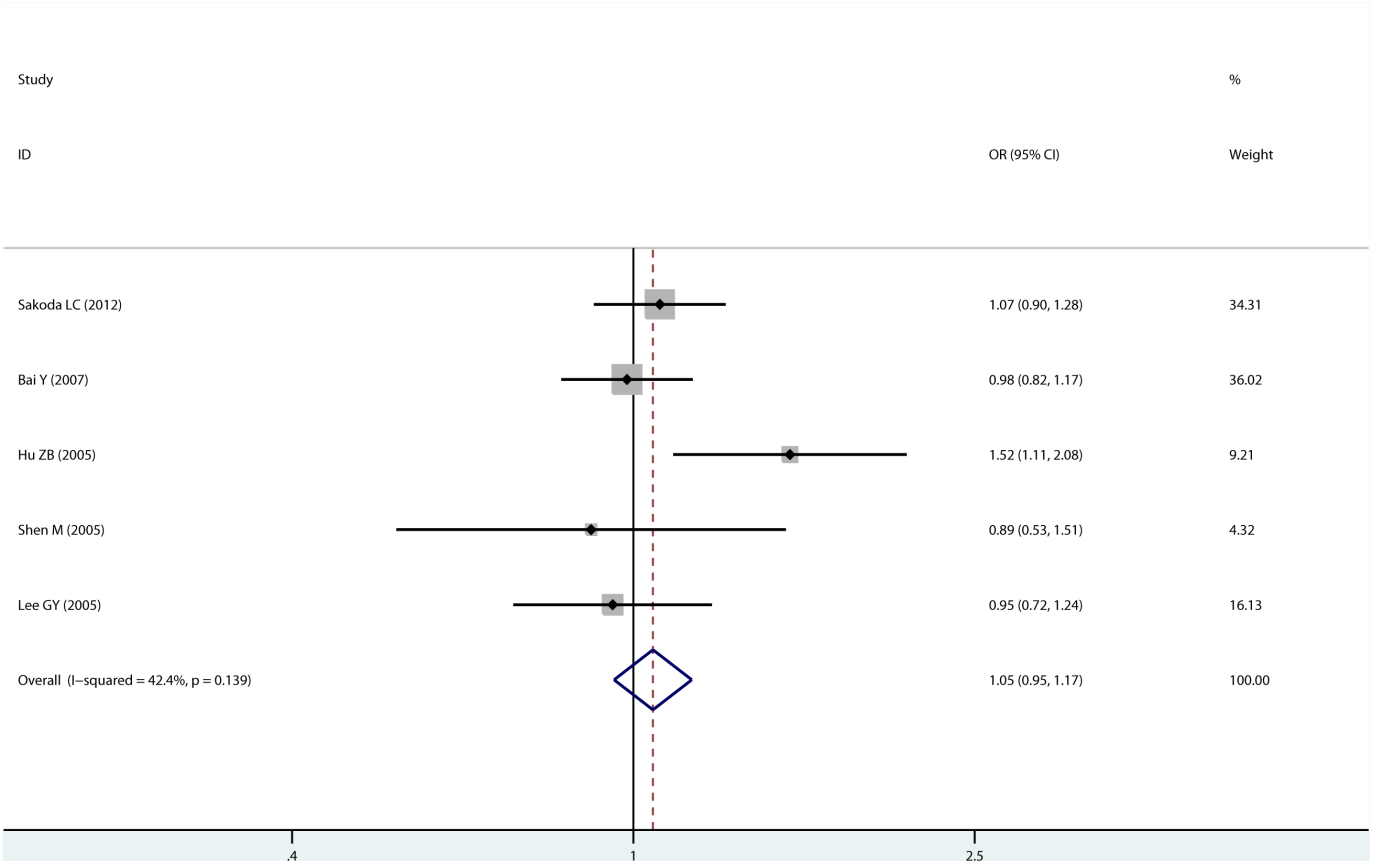
Forrest plot of XPC Ala499Val polymorphism (AlaVal/ValVal vs. AlaAla).

2605 patients and 3329 controls contributed to the analysis of XPC Ala499Val polymorphism. By pooling all eligible studies, we did not find any significant association between XPC Ala499Val polymorphism and susceptibility to lung cancer (AlaVal/ValVal, OR = 1.054, 95% CI: 0.950–1.170; Figure 3). Further stratified analysis were performed for sources of control, ethnicity and sample size, and none of these confounding factors affected the pooled results. Meta-analysis results for Ala499Val polymorphism were shown in Table 3.Begg’s test (P = 0.462, Figure S2) and Egger’s test (P = 0.762) suggested no evidence of publication bias.

**Table 3:** Meta-Analysis Results of XPC Ala499Val Polymorphism.

	Homozygote Model		Heterozygote Model		Dominant Model		Recessive Model		Allele Model	
	OR(95% CI)	Heterogeneity	OR(95% CI)	Heterogeneity	OR(95% CI)	Heterogeneity	OR(95% CI)	Heterogeneity	OR(95% CI)	Heterogeneity
Overall	1.115(0.911–1.365)	0.558	1.043(0.936–1.163)	0.14	1.054(0.950–1.170)	0.139	1.104(0.908–1.342)	0.718	1.050(0.968–1.138)	0.273
CB	1.120(0.826–1.521)	0.359	1.150(0.986–1.340)	0.922	1.144(0.987–1.326)	0.103	1.052(0.781–1.416)	0.658	1.096(0.975–1.231)	0.157
HB	1.111(0.849–1.455)	0.33	0.946(0.811–1.104)	0.146	0.972(0.838–1.126)	0.814	1.145(0.884–1.483)	0.299	1.009(0.902–1.129)	0.504
Caucasian	1.013(0.690–1.488)	NA	1.083(0.901–1.302)	NA	1.074(0.899–1.282)	NA	0.980(0.673–1.428)	NA	1.044(0.905–1.205)	NA
Asian	1.157(0.912–1.468)	0.445	1.023(0.894–1.170)	0.083	1.044(0.917–1.187)	0.076	1.154(0.918–1.450)	0.666	1.052(0.954–1.161)	0.162
Large	1.130(0.918–1.391)	0.436	1.050(0.940–1.173)	0.086	1.061(0.954–1.180)	0.088	1.115(0.912–1.362)	0.585	1.056(0.972–1.147)	0.193
Small	0.893(0.379–2.106)	NA	0.893(0.512–1.557)	NA	0.893(0.529–1.506)	NA	0.942(0.416–2.132)	NA	0.925(0.623–1.372)	NA

OR: odds ratio; CI: confidence intervals; NA: not available.

### XPC PAT Polymorphism

**Figure 4:**
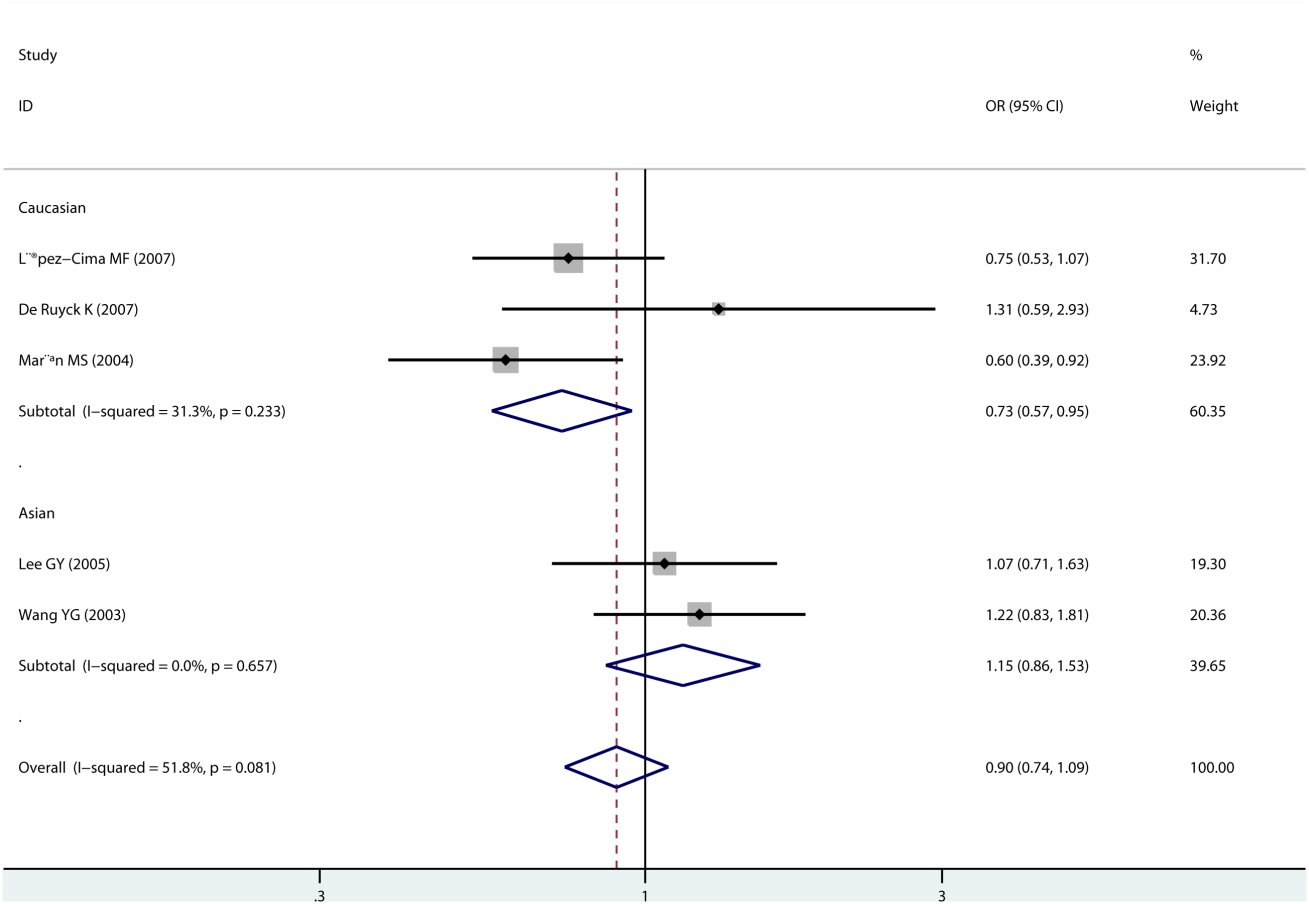
Forrest plot of XPC PAT polymorphism (−/− vs. +/+) by ethnicity.

The PAT polymorphism was investigated in 5 studies, including 2014 lung cancer patients and 1958 controls. Only homozygote comparison and heterozygote comparison models were conducted for PAT polymorphism. The overall analysis suggested that the PAT polymorphism was not significantly associated with lung cancer risk. Subgroup analysis according to sources of controls found no significant association either. While in Caucasian population, we found carriers of the −/− genotype were associated significantly reduced risk of lung cancer in homozygote comparison model (−/− vs +/+, OR = 0.735, 95% CI: 0.567–0.952; Figure 4), and the −/+ genotype was also marginally associated with reduced risk (−/+ vs ++, or = 0.786, 95% CI: 0.615–1.004). No association was observed in Asian population. In the subgroup analysis of “large” studies, results showed that the −/+ genotype reduced risk of lung cancer (−/+ vs ++, OR = 0.812, 95% CI: 0.671–0.983). Meta-analysis results for PAT polymorphism were shown in Table 4.No evidence of publication bias was found (Figure S3).

**Table 4:** Meta-Analysis Results of XPC PAT Polymorphism.

	Homozygote Model		Heterozygote Model	
	OR(95% CI)	Heterogeneity	OR(95% CI)	Heterogeneity
Overall	0.914(0.686–1.218)	0.081	0.842(0.700–1.013)	0.305
HB	0.834(0.615–1.131)	0.157	0.791(0.641–0.976)*	0.34
CB	1.223(0.826–1.809)	NA	1.037(0.706–1.522)	NA
Caucasian	0.735(0.567–0.952)*	0.233	0.786(0.615–1.004)	0.188
Asian	1.151(0.865–1.530)	0.657	0.922(0.696–1.221)	0.377
Large	0.879(0.722–1.071)	0.06	0.812(0.671–0.983)*	0.456
Small	1.313(0.589–2.926)	NA	1.461(0.691–3.089)	NA

OR: odds ratio; CI: confidence intervals; NA: not available; *significant association.

## Discussion

XPC is one of the 8 key genes in the NER pathway and is involved in the damage recognition, open complex formation and reparation [27]. The NER pathway is primarily responsible for eliminating a wide variety of DNA lesions, and thus is an important defense mechanism against structurally unrelated DNA lesions[17]. Previous studies have suggested that DNA adduct levels can predict lung cancer development[28]. Functional SNPs in protein coding regions may alter amino acids sequence and even protein function. The XPC Lys939Gln and Ala499Val polymorphisms occur in the protein coding regions and cause amino acids substitution in functional domain, thus it is reasonable that the functional polymorphisms of XPC (Lys939Gln, Ala499Val, and PAT) will alter DNA repair capacity and susceptibility to lung cancer.

In this meta-analysis, we identified 14 eligible studies, including 5647 lung cancer cases and 6908 controls, and analyzed the relationship between XPC Lys939Gln, Ala499Val, and PAT polymorphisms and susceptibility to lung cancer. We found that the XPC Lys939Gln, Ala499Val, and PAT polymorphisms were not associated with lung cancer risk in overall population. While the XPC Gln939Gln genotype was associated with significantly increased risk of lung cancer in Asian population and in Caucasian population, the PAT −/− genotype significantly reduced lung cancer risk.

The XPC Lys939Gln polymorphism is located in the interaction domain with TFIIH. By pooling 9 eligible studies, we found the Lys939Gln polymorphism was only correlated with increased risk of lung cancer in Asians. As for the PAT polymorphism, the significantly reduced susceptibility was only observed in Caucasians. These results suggested the existence of ethnic difference, which may caused by different genetic background, environment exposure, living style and other factors. To achieve more precise correlation, future studies should take ethnic difference in to consideration. In this meta-analysis, no obvious publication bias was detected. Additionally, no significant heterogeneity was present in most comparisons, except for several subgroups.

XPC polymorphisms and cancer risk has been investigated by several meta-analyses[11], [12]. Recently, He J and colleagues[11] performed a comprehensive meta-analysis about XPC Lys939Gln and Ala499Val polymorphisms and cancer susceptibility. Compared with He J’s work, we only focused on the association of XPC polymorphisms with lung cancer, while He J and colleagues[11] analyzed a variety of cancers, including lung cancer, breast cancer, bladder cancer, colorectal cancer, etc[11]. On the other hand, we also analyzed the PAT polymorphism. Additionally, we identified more eligible studies and performed detailed subgroup analyses. Compared with another recent meta-analysis about XPC polymorphisms and lung cancer risk reported by Liu C et al[12], we excluded the duplicate study reported by Hu Z et al[26], whereas Liu and colleagues did not excluded this study. For PAT polymorphism, Liu found that compared with −/− genotype, the PAT−/+ genotype was significantly associated with reduced risk of lung cancer in Asian population[12]. However, we found in Caucasian population that carriers of the PAT −/− genotype had significantly reduced susceptibility to lung cancer compared with carriers of the +/+ genotype, and no significant association was observed in Asian population. While the present meta-analysis is under review, Zhu ML [29] reported another meta-analysis about XPC polymorphism and lung cancer risk. Zhu ML and colleagues evaluated the Lys939Gln and Ala499Val polymorphisms, while they did not analyze the relationship between PAT polymorphism and lung cancer.

Limitations of this meta-analysis should also be highlighted. Smoking is a predominant risk factor of lung cancer and a common cause of DNA damage. Since meta-analysis is a method based on published studies, and collecting data from published studies would miss a lot of individual data. Without enough individual data, we could not determine the interaction between XPC polymorphisms and smoking. On the other hand, the criteria for selecting subjects in each eligible studies were also quite heterogeneous. For example, in the study of Sakoda LC[9], both lung cancer cases and controls were selected from the β-Carotene and Retinol Efficacy Trial, and they were all at high risk of cardiovascular risk. Lee GY (24) and colleagues chosen healthy volunteers as control, while controls in the study reported by López-Cima MF (18) were patients admitted to certain hospitals with various diagnoses. The heterogeneous criteria might also lead to potential bias. Cancer is a complex process involved both genetic and environmental factors, and the exposure to environment factors was not analyzed in this meta-analysis due to limited individual data. Additionally, the number of studies for the analysis of XPC Ala499Val and PAT polymorphisms were relatively small.

To summary, in this meta-analysis we found that Gln939Gln genotype was associated with significantly increased risk of lung cancer in Asian population but not in Caucasians; the PAT −/− genotype significantly reduced susceptibility to lung cancer in Caucasian population but not in Asians; the XPC Ala499Val polymorphism was not associated with lung cancer risk. Further studies are warranted to validated these findings.

## Supporting Information

Figure S1 **Funnel plot of XPC Lys939Gln polymorphism.** Circles represent the weight of each study.
(TIF)

Figure S2 **Funnel plot of XPC Ala499Val polymorphism.** Circles represent the weight of each study.
(TIF)

Figure S3 **Funnel plot of XPC PAT polymorphism.** Circles represent the weight of each study.
(TIF)

Checklist S1 **PRISMA Checklist.**
(DOC)
