# Supplementary figures and images for: Association of XPC Polymorphisms and Lung Cancer Risk: A Meta-Analysis
Source: PLoS One. 2014 Apr 15;9(4):e93937. doi: 10.1371/journal.pone.0093937 (PMC3988015; doi:10.1371/journal.pone.0093937)

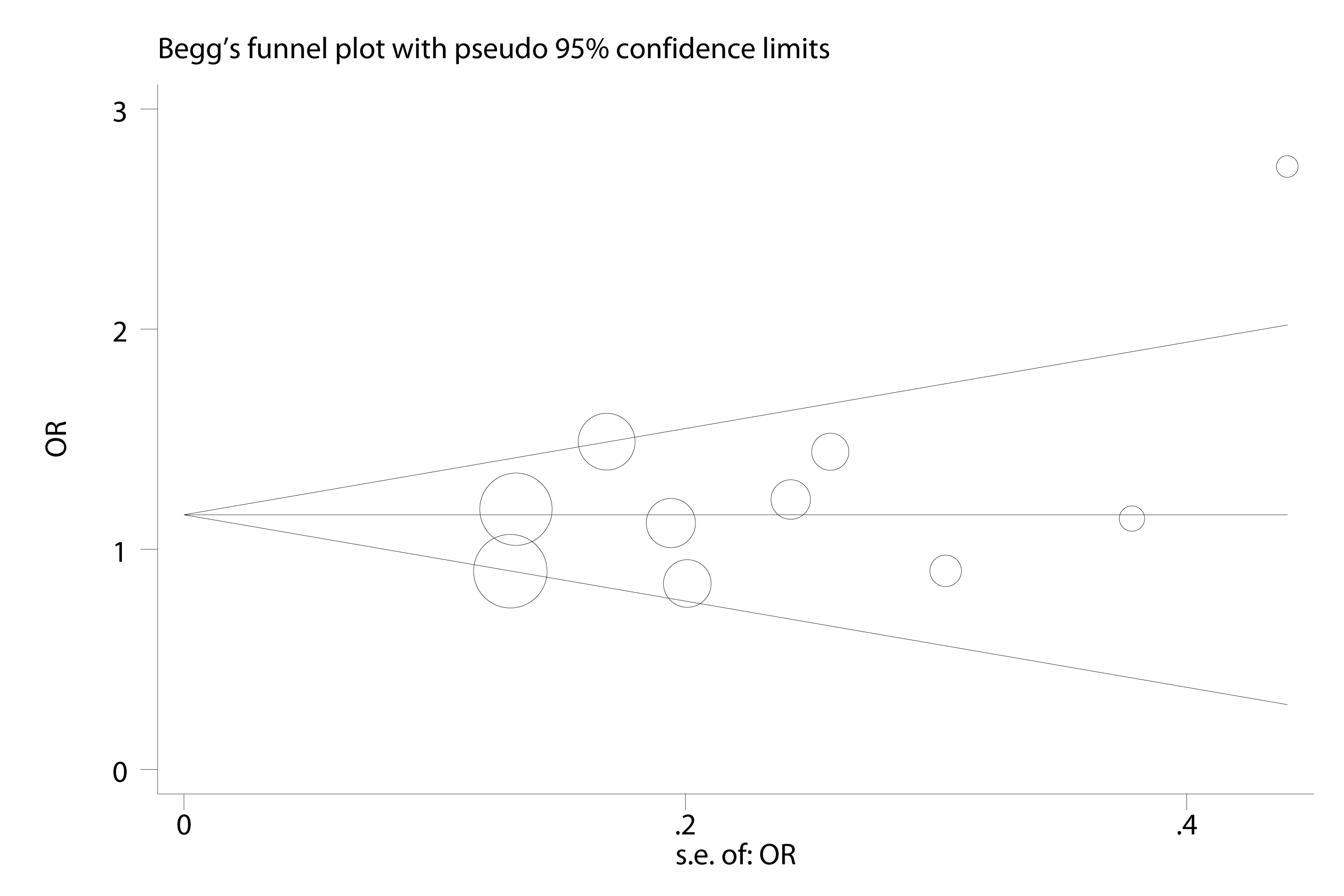

Supplement: Figure S1 — Funnel plot of XPC Lys939Gln polymorphism. Circles represent the weight of each study. (TIF) [file pone.0093937.s001.tif]

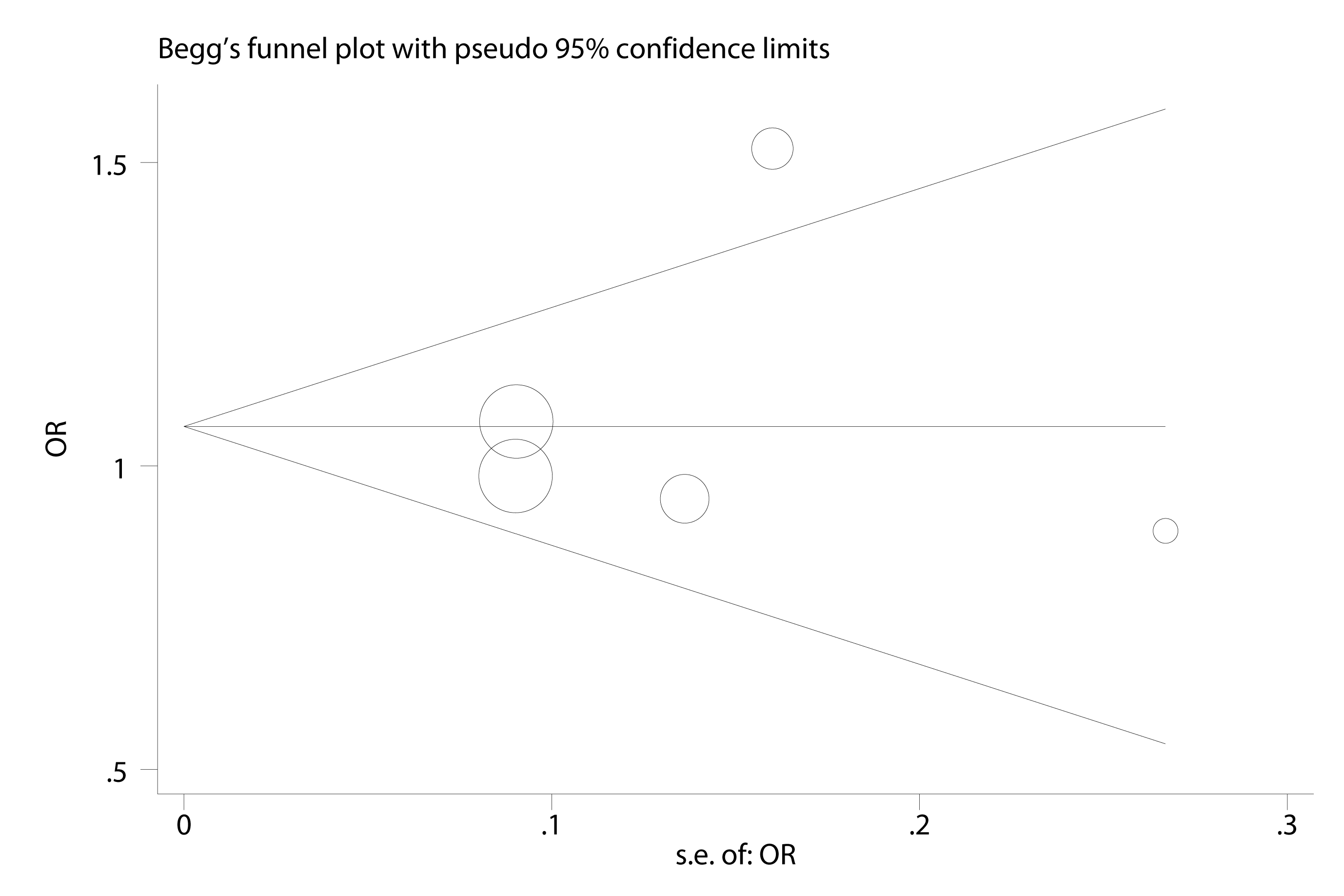

Supplement: Figure S2 — Funnel plot of XPC Ala499Val polymorphism. Circles represent the weight of each study. (TIF) [file pone.0093937.s002.tif]

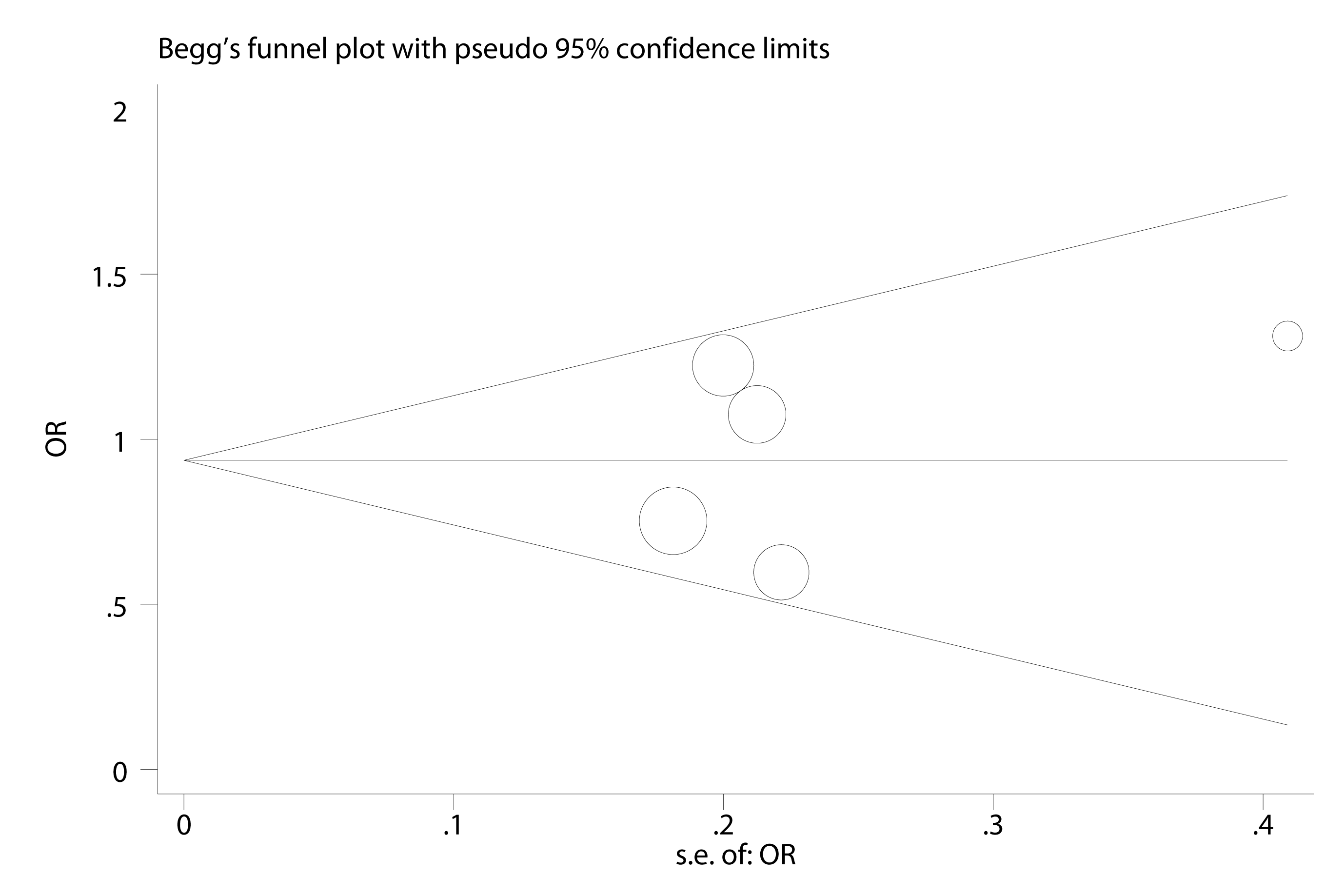

Supplement: Figure S3 — Funnel plot of XPC PAT polymorphism. Circles represent the weight of each study. (TIF) [file pone.0093937.s003.tif]
